# Association between demarcated opacities in primary canines and hypomineralized second primary molars

**DOI:** 10.1590/1807-3107bor-2026.vol40.080

**Published:** 2026-10-23

**Authors:** Mylenne Karolayne Silva Ribeiro, Lavínia Lourenço Costa, Luisa Madeira Lemos, Jaderson Felipe Santos Dantas, Patrícia Bittencourt Santos

**Affiliations:** (a) Universidade Federal do Rio Grande do Norte – UFRN, Graduate Program in Dental Sciences, Department of Dentistry, Natal, RN, Brazil.; (b) Universidade Federal do Rio Grande do Norte – UFRN, Department of Dentistry, Natal, RN, Brazil.

**Keywords:** Dental Enamel Hypomineralization, Tooth Abnormalities, Tooth, Deciduous

## Abstract

To evaluate the association between the presence of demarcated opacities in primary canines and the occurrence of hypomineralized second primary molars (HSPM) in a group of Brazilian children. Dental records from 232 patients aged 5–10 years were evaluated by a single trained and calibrated examiner using the diagnostic criteria of the European Academy of Paediatric Dentistry (EAPD). The presence, severity, and clinical characteristics of enamel defects were recorded. Data were analyzed using descriptive statistics, Fisher's exact test, and binomial logistic regression to assess the association between primary canine involvement (exposure) and HSPM (outcome). The prevalence of HSPM was 20.7% (48/232 children) at the patient level and 15.8% (147/928 teeth) at the tooth level, with no significant differences according to sex or age. Among affected children, 68.8% presented mild lesions, whereas 31.2% had at least one severely affected molar. Demarcated opacities were the most common clinical presentation (76.9%). Logistic regression revealed that each additional affected primary canine was associated with a 6.12-fold increase in the odds of HSPM (OR = 6.12; 95%CI: 1.79–20.96; Nagelkerke R^2^ = 0.145). There was an association between demarcated opacities in primary canines and HSPM. Involvement of primary canines may serve as a clinical marker for the occurrence of HSPM, supporting the implementation of early screening and preventive strategies.

## Introduction

Hypomineralized second primary molars (HSPM) constitute a developmental enamel defect characterized by qualitative changes in enamel translucency and a multifactorial etiology. The condition affects at least one second primary molar and may also involve other primary teeth, particularly canines.^1^ Clinically, well-demarcated white, yellow, or brown opacities may be observed and can be associated with post-eruptive breakdown (PEB), atypical carious lesions, and atypical restorations.^2^

Affected enamel exhibits reduced mechanical strength because of its high porosity, making it susceptible to fracture even under normal masticatory forces.^3^ Enamel loss exposes the underlying dentin, thereby increasing the risk of dentin hypersensitivity, biofilm accumulation, and carious lesion development. Consequently, HSPM may substantially affect children's quality of life, resulting in limitations during daily activities such as tooth brushing and chewing.^4^

The etiology of HSPM is considered multifactorial but remains inconclusive.^5^ The literature reports associations between HSPM and several potential prenatal, perinatal, and postnatal factors, including maternal alcohol and tobacco consumption, maternal hypertension, low birth weight, prematurity, delivery complications, neonatal incubation, lack of breastfeeding, antibiotic use during childhood, early childhood fever, and asthma.^6^

The first study investigating the prevalence of HSPM was conducted by Elfrink et al.^1^ in the Netherlands and reported a prevalence of 4.9%. However, reported prevalence rates vary considerably in the literature, making comparisons difficult because of differences in diagnostic criteria.^7^ A recent meta-analysis estimated the global prevalence of HSPM at 6.8%.^8^ Over the past decade, developmental enamel defects have gained considerable attention in both clinical and scientific settings because of their substantial impact on children's oral health.^9^

Despite the available prevalence data, evidence regarding the distribution patterns of HSPM throughout the primary dentition remains limited. In particular, it is unclear whether enamel defects in primary canines are associated with defects in teeth mineralizing during the same developmental period, such as second primary molars. Accordingly, this study aimed to evaluate the association between the presence of demarcated opacities in primary canines and the occurrence of HSPM in a group of Brazilian children.

## Methods

### Study design

This cross-sectional study was conducted in the city of Natal, Brazil, and followed the Strengthening the Reporting of Observational Studies in Epidemiology (STROBE) guidelines. Ethical approval was obtained from the Research Ethics Committee of the Universidade Estadual do Rio Grande do Norte. The study complied with the ethical principles established by the Council for International Organizations of Medical Sciences (CIOMS) for research involving clinical records.^10^

### Setting and participants

The sample size was calculated using data from a pilot study involving the dental records of 50 patients, which identified an HSPM prevalence of 15.6%. Using this estimate, a confidence level of 95%, and a margin of error of 5%, the minimum required sample size was calculated as 203 patients.

Dental records of patients aged 5–10 years who received care at the Universidade Estadual do Rio Grande do Norte between 2018 and 2024 were reviewed. Each record included panoramic radiographs, intraoral photographs, and medical history information. Five standardized intraoral photographs were available for each patient, comprising maxillary and mandibular occlusal views, right and left lateral views, and a frontal view. All photographs were stored in high-quality JPEG format and were obtained using standardized imaging protocols and equipment settings.

Children with four erupted second primary molars and primary canines were included. Cases in which second primary molars were absent were also included when those teeth would be expected to be present based on the child's age. Extractions were attributed to HSPM when they were associated with defects in other second primary molars, a history of low caries risk, and age-appropriateness. Exclusion criteria included patients undergoing orthodontic treatment, images that were blurred, dark, or pixelated, or did not allow full visualization of all tooth surfaces.

### Variables

The dependent variable was the occurrence of HSPM. The primary independent variable was the presence of demarcated opacities in primary canines. Additional independent variables included the number of affected canines, age, and sex.

### Data collection

#### HSPM

The diagnosis of HSPM was based on criteria established by the European Academy of Paediatric Dentistry (EAPD).^11^ These criteria include white, yellow, or brown demarcated opacities, PEB associated with opacities, atypical restorations, and extractions due to HSPM. The distribution of hypomineralized defects was recorded, and lesions were classified according to severity. Mild lesions were characterized by demarcated opacities without enamel loss, whereas severe lesions exhibited PEB, atypical carious lesions, atypical restorations, or extractions. A child was classified as having severe HSPM when at least one second primary molar exhibited severe hypomineralization.

#### Bias

All photographs were evaluated by a single calibrated examiner according to EAPD criteria and the protocol proposed by Ghanim et al.^12^ The calibration process included comprehensive training, detailed discussions of diagnostic criteria, the evaluation of a set of 50 photographs containing healthy teeth and various developmental enamel defects, including HSPM.

To assess diagnostic consistency, the examiner re-evaluated the same set of photographs one week after the initial assessment. Calibration was validated by a pediatric dentist who served as the reference standard. High interexaminer and intraexaminer agreement was confirmed by kappa coefficients of 0.88 and 0.91, respectively, indicating excellent reliability.

### Statistical analysis

Data were analyzed using Jamovi software (version 1.6). Descriptive statistics were used to characterize the sample and summarize the EAPD diagnostic criteria at the tooth level. Pearson's chi-square test and Fisher's exact test were used to assess associations between variables. Binomial logistic regression was performed to evaluate the association between primary canine involvement and the occurrence of HSPM. Statistical significance was set at p < 0.05.

## Results

### Prevalence

Among the 232 dental records evaluated, HSPM was diagnosed in 48 children (20.7%), corresponding to a tooth-level prevalence of 15.8%. No statistically significant differences were observed between boys and girls (X^2^ = 0.799, p = 0.371). Demographic characteristics are presented in Table 1.

**Table 1:** Distribution of sex and age among participating children (n = 232 children).

Variable		HSPM			p-value*
		Present	Absent	Total	
		n (%)	n (%)	n (%)	
Sex					
	Male	23 (23.5)	75 (76.5)	98 (100)	X^2^ = 0.799
	Female	25 (18.7)	109 (81.3)	134 (100)	p = 0.371
Age (Years)					
	5	1 (9.1)	10 (90.9)	11 (100)	
	6	2 (7.4)	25 (92.6)	27 (100)	
	7	16 (29.6)	38 (70.4)	54 (100)	
	8	12 (23.5)	39 (76.5)	51 (100)	
	9	11 (19.6)	45 (80.4)	56 (100)	
	10	6 (18.2)	27 (81.8)	33 (100)	

HSPM: hypomineralized second primary molars.

* Results based on Pearson's chi-square test.

#### Distribution of HSPM severity

Most affected children presented mild lesions, whereas nearly one-third had at least one severely affected molar. Table 2 shows the distribution of mild and severe HSPM across age groups. Primary canine involvement was observed in 4.3% of the total sample. All affected canines exhibited mild lesions characterized exclusively by white-cream demarcated opacities, with no evidence of PEB or atypical restorations (Figure).

**Table 2:** Distribution of HSPM severity according to age at the patient level (n = 48 children with HSPM).

Variable	HSPM			p-value*
Age (Years)	Mild	Severe	Total	
	n (%)	n (%)	n (%)	
5	0 (0)	1 (100)	1 (100)	0.435
6	2 (100)	0 (0)	2 (100)	
7	13 (81.3)	3 (18.7)	16 (100)	
8	8 (66.7)	4 (33.3)	12 (100)	
9	6 (54.5)	5 (45.5)	11 (100)	
10	4 (66.7)	2 (33.3)	6 (100)	

HSPM: hypomineralized second primary molars.

* Results based on Fisher's exact test.

**Figure:**
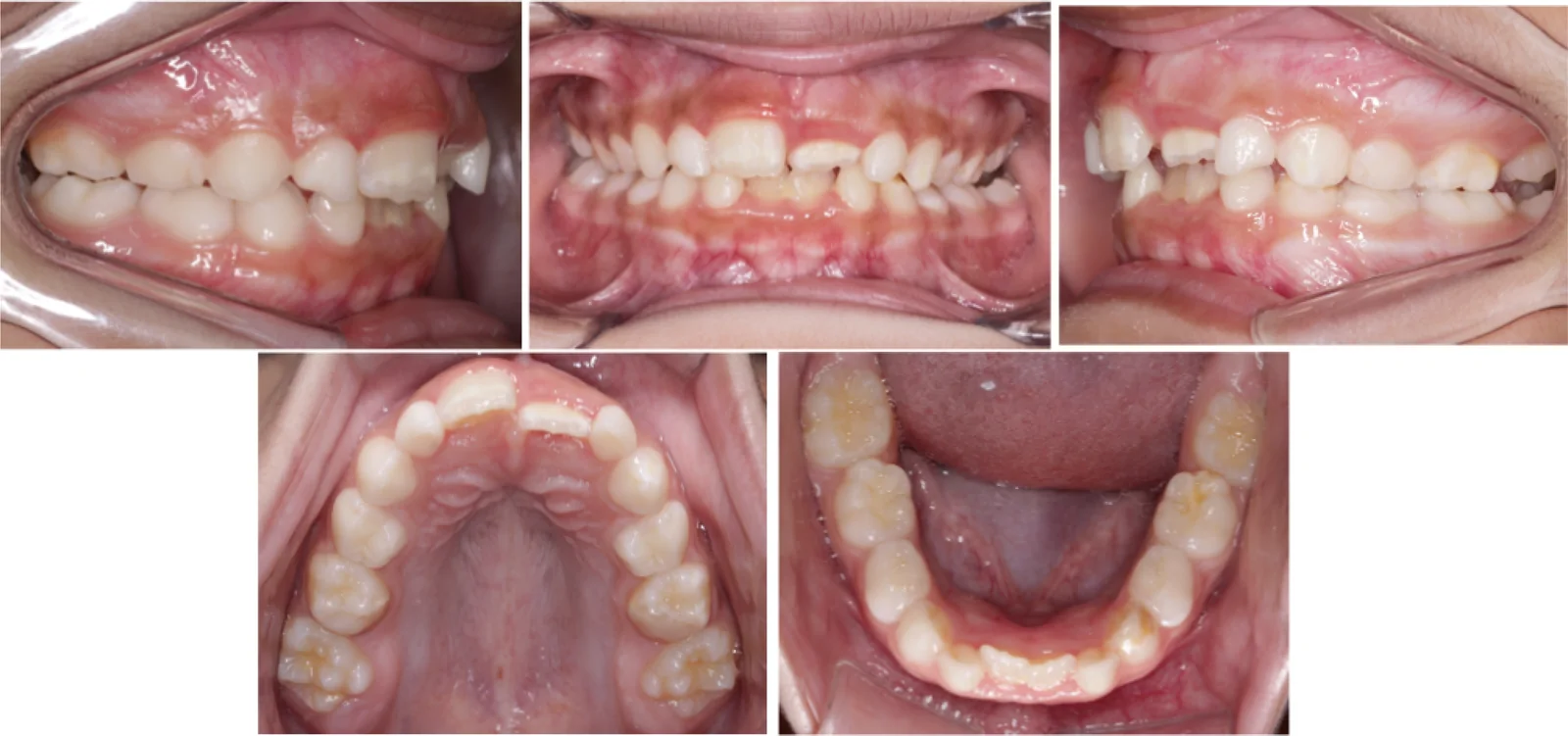
Clinical presentation of enamel defects in the primary dentition.

#### Clinical characteristics of HSPM at the tooth level

Table 3 presents the clinical status of HSPM at the tooth level according to the EAPD criteria. Among the 928 second primary molars evaluated, 147 (15.8%) exhibited HSPM. Demarcated opacities (76.9%) were the most common clinical presentation, followed by PEB (11.6%) and atypical restorations (9.5%). A significant association was identified between the presence of demarcated opacities in primary canines and the diagnosis of HSPM (p < 0.001). Children with affected primary canines exhibited a 4.10-fold higher prevalence of HSPM compared with those without canine involvement (PR = 4.10; 95%CI: 1.18–14.2) (Table 4). Table 5 presents the results of the logistic regression analysis, which confirmed primary canine involvement as a significant clinical marker of HSPM (p = 0.004). Each additional affected primary canine was associated with a 6.12-fold increase in the odds of HSPM (OR = 6.12; 95%CI: 1.79–20.96). The model explained 14.5% of the variance in HSPM occurrence.

**Table 3:** Distribution of HSPM characteristics according to EAPD criteria at the tooth level (n = 147 second primary molars).

Tooth	Demarcated opacities	PEB	Atypical restoration	Atypical caries	Extracted	Total
	n (%)					
55	30 (75.0)	5 (12.5)	3 (7.5)	1 (2.5)	1 (2.5)	40 (100)
65	34 (85.0)	4 (10.0)	2 (5.0)	0 (0)	0 (0)	40 (100)
75	25 (73.5)	5 (14.7)	3 (8.8)	0 (0)	1 (2.9)	34 (100)
85	24 (72.7)	3 (9.1)	6 (18.2)	0 (0)	0 (0)	33 (100)
Total	113 (76.9)	17 (11.6)	14 (9.5)	1 (0.7)	2 (1.4)	147 (100)

PEB: post-eruptive breakdown; HSPM: hypomineralized second primary molars.

**Table 4:** Association between the presence of demarcated opacities in primary canines and HSPM (n = 232 children).

Opacity in primary canine	HSPM		Total	PR	95%CI	p-value*
	Yes	No				
	n (%)					
Yes	8 (80)	2 (20)	10 (100)	4.10	1.18–14.2	< 0.001*
No	40 (18)	182 (82)	222 (100)			

HSPM: hypomineralized second primary molars; CI: confidence interval; PR: prevalence ratio.

* Results based on Fisher's exact test.

**Table 5:** Logistic regression analysis evaluating the association between the number of affected primary canines and HSPM (n = 232 children).

Variable	OR	p-value*	95%CI	R^2^_N_
Number of affected primary canines	6.12	0.004	1.79–20.96	0.145

OR: odds ratio; CI: confidence interval; R^2^_N:_ Nagelkerke's R^2^.

* Results based on logistic regression.

## Discussion

The present study evaluated the association between demarcated opacities in primary canines and the occurrence of HSPM in children aged 5–10 years. Overall, the findings demonstrated a strong association between these enamel defects. This relationship is biologically plausible because crown mineralization of primary second molars and primary canines occurs within a similar developmental period.^13^ This overlap extends approximately from the 18th week of intrauterine life to the first 9 months after birth, during which amelogenesis is particularly susceptible to systemic disturbances.^14^ Adverse conditions occurring during this critical developmental window, including maternal illness, perinatal complications, nutritional deficiencies, and early childhood diseases, may interfere with enamel formation and result in the concomitant occurrence of hypomineralization in multiple teeth.^15^

Furthermore, the logistic regression model demonstrated that each additional affected primary canine was associated with a 6.12-fold increase in the odds of HSPM. This finding may reflect the extent of the underlying systemic disturbance, suggesting that children with a greater number of affected canines are more likely to exhibit defects in other teeth mineralizing during the same developmental period^16^. However, the variance explained by the model (Nagelkerke R^2^ = 0.145) indicates that a substantial proportion of the variability in HSPM occurrence remains unexplained. This finding highlights the likely influence of additional unmeasured factors, consistent with the multifactorial nature of HSPM. The condition is thought to result from the interaction of systemic and genetic factors, making it challenging to isolate a single cause or fully predict its occurrence through clinical markers alone.

These findings are consistent with previous studies indicating that enamel defects can extend beyond the index teeth affected by molar-incisor hypomineralization (MIH) and HSPM.^17,18^ Primary canines erupt earlier and occupy a more anterior and visible position in the dental arch. Therefore, they may serve as clinical indicators for the early identification of HSPM during routine examinations, particularly in public health settings. In this context, it is important to recognize that hypomineralization may manifest differently according to tooth type. Whereas molars are more frequently affected by moderate and severe defects, canines typically exhibit milder lesions, often limited to demarcated opacities on the buccal surface.^14^ Surfaces subjected to masticatory forces are more susceptible to PEB, which represents a more severe manifestation of enamel hypomineralization.^19^ Differences in clinical presentation further support the rationale that early detection of enamel defects in primary canines may help identify children at increased risk of more severe hypomineralization patterns affecting second primary molars.

The prevalence of HSPM observed in the present study was 20.7%, exceeding the global estimate of 6.8% reported by McCarra but remaining within the broad range reported in the literature (0–41%).^8,20,21^ These differences may be attributable to variations in diagnostic criteria, age ranges, and environmental or genetic factors specific to each population.^22^

Brazilian studies investigating HSPM prevalence remain limited and have reported rates ranging from 6.48%^14^ to 16.8%,^23^ with values as high as 24.4% reported in more recent investigations.^24^ This variation likely reflects the continental dimensions of Brazil, marked socioeconomic disparities, and population heterogeneity resulting from extensive genetic admixture.^25,26^ These findings underscore the challenges associated with comparing HSPM prevalence across populations and highlight the importance of considering regional and demographic characteristics when interpreting epidemiologic data.

At the tooth level, the prevalence of HSPM was 15.8%. This finding is consistent with previous reports,^8,27^ indicating that HSPM does not affect all second primary molars uniformly.^20^ Such variability supports the hypothesis that HSPM has a systemic origin but exhibits an asymmetric pattern of expression.^18^ Consistent with previous studies, no sex-related differences in HSPM occurrence were observed.^20,28^ These findings are important for promoting equitable approaches to pediatric dental care and ensuring that all children receive appropriate preventive and therapeutic interventions.^20^

The prevalence of HSPM did not differ significantly across the age groups evaluated. Although some studies have reported a decline in the detectability of enamel defects with increasing age, primarily because of masking by carious lesions, extensive restorations, or tooth loss,^6,8^ HSPM was identified consistently throughout the 5–10-year age range. This finding may be attributable to the diagnostic criteria adopted, which included atypical restorations and extractions attributed to HSPM, thereby reducing the likelihood of underestimating prevalence among older children.

Demarcated opacities were the most common clinical manifestation, followed by PEB and atypical restorations. This pattern is consistent with findings reported across different geographic regions and reinforces the role of demarcated opacities as the hallmark clinical feature of HSPM.^18^ Severe manifestations were observed in 31.2% of the affected children and are particularly concerning because they are associated with increased hypersensitivity and a greater risk of carious lesion development, thereby adversely affecting children's quality of life.^4^

The use of standardized, high-resolution photographs is a validated method for assessing enamel defects, demonstrating high agreement with clinical examinations while allowing for blinded and objective reassessment. However, this approach may introduce diagnostic uncertainty and potentially overestimate defect frequency, since opacities may be difficult to distinguish from early carious lesions in retrospective records. In addition, attributing extractions to HSPM based on indirect criteria requires caution because treatment decisions in clinical practice are often multifactorial. Despite these inherent limitations, the use of EAPD criteria and rigorous examiner calibration helped minimize observational bias and support the reliability of the associations identified.

Finally, this study has some limitations. Data were obtained from a single dental center, which may limit the generalizability of the findings to other regions, particularly considering the marked regional and socioeconomic diversity of Brazil. Environmental exposures, nutritional status, and other population characteristics vary throughout the country and may influence the prevalence of enamel defects. In addition, although the calculated sample size was achieved, the cross-sectional design precludes causal inference. Therefore, the findings should be interpreted as associations rather than cause-and-effect relationships. Future longitudinal multicenter studies are warranted to confirm these patterns in more diverse populations.

## Conclusions

The prevalence of HSPM in this sample of Brazilian children was 20.7% at the patient level and 15.8% at the tooth level. A significant association was observed between the presence of demarcated opacities in primary canines and HSPM. Each additional affected primary canine was associated with a 6.12-fold increase in the odds of HSPM. These findings suggest that identifying enamel defects in earlier-erupting primary canines may help clinicians recognize children at increased risk of HSPM and support the use of primary canines as an early clinical marker of the condition.

## Data Availability

The datasets generated during and/or analyzed during the current study are available from the corresponding author on reasonable request.
